# Like a Rolling Stone: Sting-Cgas Pathway and Cell-Free DNA as Biomarkers for Combinatorial Immunotherapy

**DOI:** 10.3390/pharmaceutics12080758

**Published:** 2020-08-11

**Authors:** Guillaume Sicard, Frédéric Fina, Raphaelle Fanciullino, Fabrice Barlesi, Joseph Ciccolini

**Affiliations:** 1 SMARTc Unit, CRCM Inserm U1068, Aix Marseille University, 13007 Marseille, France; guillaume.sicard@ap-hm.fr (G.S.); raphaelle.fanciullino@univ-amu.fr (R.F.); 2 Anatomo-pathology Unit, La Timone University Hospital of Marseille, 13005 Marseille, France; frederic.fina@ap-hm.fr; 3 School of Medicine, Aix Marseille University, 13007 Marseille, France; Fabrice.BARLESI@gustaveroussy.fr; 4 Gustave Roussy Institute, 94800 Villejuif, France

**Keywords:** combinatorial immunotherapy, cytotoxics, biomarkers, precision medicine

## Abstract

Combining immune checkpoint inhibitors with other treatments likely to harness tumor immunity is a rising strategy in oncology. The exact modalities of such a combinatorial regimen are yet to be defined, and most attempts have relied so far on concomitant dosing, rather than sequential or phased administration. Because immunomodulating features are likely to be time-, dose-, and-schedule dependent, the need for biomarkers providing real-time information is critical to better define the optimal time-window to combine immune checkpoint inhibitors with other drugs. In this review, we present the various putative markers that have been investigated as predictive tools with immune checkpoint inhibitors and could be used to help further combining treatments. Whereas none of the current biomarkers, such as the PDL1 expression of a tumor mutational burden, is suitable to identify the best way to combine treatments, monitoring circulating tumor DNA is a promising strategy, in particular to check whether the STING-cGAS pathway has been activated by cytotoxics. As such, circulating tumor DNA could help defining the best time-window to administrate immune checkpoint inhibitors after that cytotoxics have been given.

## 1. Introduction: Doom and Gloom

Precision medicine is a broad generic term, generally used to describe all of the resources used to decipher the molecular and genomic profiles of tumors, primarily for selecting the drugs which are the most likely to be clinically effective in cancer patients.

For decades, improving either response rates or survival in cancer patients has been achieved in a stepwise manner. Despite the recent and growing use of bio-guided medicine in oncology, the use of targeted therapies or anti-angiogenic therapy has been considered as an incremental innovation, with a significant but moderate impact on survival eventually. Indeed, apart from rare counter-examples such as imatinib in CML or trastuzumab in HER2-positive breast cancer, it has taken decades to reach meaningful prolonged survival, beyond a rapid and spectacular increase in response rates [1].

More recently, immunotherapy has been considered as a ground-breaking innovation in oncology. Although the role of tumor immunity has been known for decades [2], the introduction of immune checkpoint inhibitors (e.g., anti-CTLA4, or PD1/PDL1 axis antagonists) as new anticancer agents quickly led to spectacular improvements in clinical outcomes, including 5 year-survival rates above 30%. Unfortunately, successful immunotherapy is restricted to a too limited, but relevant, number of cancers with once dismal prognosis, such as metastatic melanoma and advanced non-small cell lung cancer (NSCLC).

Consequently, after first years of use as single-agents, all the newly developed immune checkpoint inhibitors are to be combined with other therapeutic strategies such as chemotherapy, targeted therapy, anti-angiogenics, or radiation therapy. Importantly, most combinational trials have been designed thus far on an empirical basis, i.e., by adding novel immune checkpoint inhibitors to already existing and sometimes decade-old regimens. Still, today a rising amount of evidence suggests that to achieve a maximum efficacy while controlling toxicities, there is probably an optimal way immune checkpoint inhibitors should be combined with other anticancer drugs or with radiation therapy [3]. Of note, this kind of optimal design cannot be identified anymore with standard trial-and-error approaches, owing to the ever growing complexity and the countless possibilities of different combinations to be tested [4].

Academic clinical studies have already shown, with other anticancer agents such as anti-angiogenics and cytotoxics, that optimizing administration scheduling can help improving clinical outcomes. This, calls for new methods to administrate drugs and determine the right dosing, scheduling, and sequencing when setting up a combinatorial regimen [5]. 

It should be noted that clinical evidence establishing pharmacokinetic/pharmacodynamic (PK/PD) relationships (e.g., drug exposure levels predict survival and are associated with treatment-related toxicities) with anti-CTLA4 Ipilumumab have been published, as well as high inter-patient variability in pharmacokinetic profiles [6]. With other immune checkpoint inhibitors targeting the PD1/PDL1 axis, exposure–effects relationships are not fully elucidated yet since conflictual results have been published so far, such as with anti-PD1 nivolumab [7,8].

**Figure 1:**
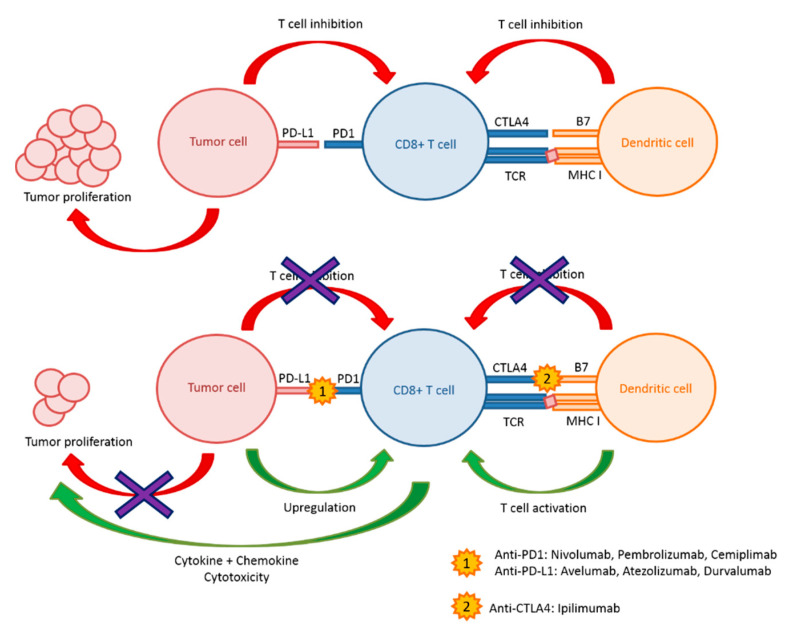
Schematic representation of how immune checkpoint inhibitors work.

As their name suggests, immune checkpoint inhibitors inhibit the key checkpoints of immune response [9]. Tumor cells have the ability to escape from host immunity [10] and the role of immune checkpoint inhibitors is to target transmembrane proteins such as the CTLA4, PD1, and PD-L1 ligand. Currently, the 7 immune checkpoint inhibitors which have been FDA-approved are [11] anti-CTLA4 (Ipilimumab), Anti-PD1 (Nivolumab, Pembrolizumab, Cemiplimab), and Anti-PD-L1 (Avelumab, Atezolizumab, Durvalumab). Many others immune checkpoint inhibitors are currently being developed and are already in clinical testing phases [12]. Mechanisms of action of immune checkpoint inhibitors are briefly summarized in Figure 1.

Immune checkpoint inhibitors are used in current practice in oncology as single agent, and more and more frequently now in combination with chemotherapy, radiation therapy, or in association with another immune checkpoint inhibitor such as the anti-CTLA4 + anti-PD1 combo in metastatic melanoma [13].

The first FDA-approved immune checkpoint inhibitor was Ipilimumab in the early 2010’s [14] in advanced melanoma. Ipilimumab is a fully human monoclonal antibody that targets and binds to cytotoxic T-lymphocyte-associated antigen 4 (CTLA4) and blocks its interaction with its ligands, i.e., CD80 and CD86. Consequently, Ipilimumab potentiates the antitumor T-cell response, resulting in unrestrained T-cell proliferation, plus it provides co-stimulatory signals (CD80/CD86) through CD28 on T lymphocytes. CTLA4 is constitutively expressed on the lymphocytes T regulatory (best known as T Regs) surface. The role of CTLA4 is an inhibitory function, which impedes acquisition of T cell effector function, thus preventing immunity from going out of control.

Anti-PD1 monoclonal antibodies Nivolumab and Pembrolizumab have been approved in 2014, and Cemiplimab in 2018. Anti-PD-L1 monoclonal antibody Atezolizumab was approved in 2016 and Avelumab and Durvalumab in 2017. Programmed cell death protein 1 (PD1) is a transmembrane protein expressed by multiple immune cells, whereas its ligand, PD-L1 is expressed by non-blood cells and overexpressed by tumor cells [15]. The PD1/PD-L1 axis is major immunosuppressive pathway, that normally helps to control immune reactions. This axis leads to the reduced proliferation of T Lymphocytes, anergy and exhaustion, apoptosis of activated T lymphocytes, diminution of T lymphocytes’ TCR-mediated activation and proliferation, plus a decrease in several cytokines such as interferon-γ (IFN-γ) and interleukin-2, as well as increase in T Regs further inhibiting immune response [16].

Regarding side-effects, immune checkpoint inhibitors are generally well tolerated drugs but can trigger severe toxicities rarely seen in oncology thus far, best known as immune-related adverse events (IRAE). The most recurrent toxicities are immune events indeed, such as skin and digestive toxicities and metabolic disorders [17]. Cardiac toxicity has been also reported with immune checkpoint inhibitors, including cases of lethal myocarditis [18]. Because some of these toxicities can thus be life-threatening, optimizing the efficacy/toxicity balance is of major interest with immunotherapy.

The use of immune checkpoint inhibitors in association with conventional chemotherapies or oral targeted therapies is a promising strategy because there are no overlapping toxicities with immunotherapy, whereas a synergistic effect is expected at the tumor level. These combinations are based upon the hypothesis that sensitivity to immune checkpoint inhibitors will be increased by immunomodulating properties of the associated drugs. For instance, Cisplatin increases activated T lymphocytes as well as their intratumoral infiltration, thus acting synergistically with immune checkpoint inhibitors [19]. However, determining the optimal modalities of administration of cisplatin so that its modulating features are maximal, is yet to be determined. 

## 2. Looking for the Ideal Biomarker in Immunotherapy: Welcome to the Jungle!

Importantly, the search for a robust, validated, and fully predictive biomarker with immunotherapy is still an ongoing story [20]. Ideally, such a biomarker could, beyond predicting clinical outcomes, help defining the best time-window for further combining treatments. This should accelerate the identification of optimal combinatorial strategies with immune checkpoint inhibitors, so as to expedite transfer to bedside practice and reduce attrition rates during clinical trials. 

The ideal biomarker should be specific to tumor cells, sensitive enough to separate cancer patients from healthy individuals, translatable (i.e., not specific of one type of cancer or patient) and easy and quick to quantify in a patient-friendly way (e.g., measured in peripheral blood, and not from tumor biopsies), in addition to being robust and reproducible.

The search for predictive biomarkers with immunotherapy is challenging, as fully understanding the underlying pharmacology of these drugs is tricky. Roughly, two categories of biomarkers can be distinguished: the ones linked to the tumor or the ones linked to the host. The principal biomarker linked to the host is the gut microbiome, plus neutrophil-to-lymphocyte ratios (NLR). Regarding a tumor biomarker, molecular target expression (e.g., PD-L1), inflammation state (e.g., Myeloid-Derived Suppressive Cells, tertiary lymphoid structures, tumor-infiltrating lymphocytes), and tumor antigen expression (e.g., tumor mutational burden, microsatellite instability) are the most frequently tested markers. However, all these markers are still characterized by inconsistencies in predictive cut-off values depending on the studies, thus lowering their implementation in routine oncology as robust decision-making tools.

## 3. Microbiome and Immunotherapy: Everything Counts

Interest on the role gut microbiota could play in the response to immune checkpoint inhibitors is increasingly growing. Microbiota seem to play a major role on T Regs expression and in the activation of T Lymphocytes indeed [21]. One of the mechanisms of action explaining the role of microbiota is stimulating the response of T lymphocytes against microbial antigens. This response may be tumor specific or may be the source of cross-reactions against tumor specific antigens. In addition, T lymphocytes with bacterial epitopes were found in the tumor microenvironment as well [22]. In parallel, some studies demonstrated the negative impact of antibiotic treatment before starting immune checkpoint inhibitor treatment [23]. Controlling antibiotic therapy is easily feasible, even if they cannot always be easily ruled out in oncology in patients with sepsis. However, checking that the patient’s microbiota is optimal for an optimal immunotherapy response remains highly challenging. Microbiota transplants are already a strategy in the treatment of *Clostridium difficile* infections, but to what extent it could be transposed to cancer patients is still currently under clinical investigation.

## 4. PD1 and PD-L1: Wish you Were Here

The expression of PD1 by immune cells and PD-L1 by tumor cells has been the first biomarker proposed in modern immunotherapy. The overexpression of PD-L1 confers a poorer prognosis across multiple tumor types, making therapeutic intervention on this immunomodulatory axis enticing. The quantification of PD-L1 appeared intuitively as an interesting biomarker of tumor sensitivity to immunotherapy, but the relevance of expression of PD-L1 alone remains debated today [24]. Furthermore, the cut-off for positivity of PD-L1 expression is yet be fully determined [25]. In addition, a meta-analysis in solid tumors demonstrated that immune checkpoint inhibitors decreased the risk of death by 34% to 100% in patients with positive PD-L1 and by 0% to up to 20% in PD-L1 negative patients [26], highlighting the complexity of using PD-L1 expression as a biomarker. About 10 PD-L1 immunohistochemical diagnostic assays are currently on the market or in development [27]. A study, comparing four different assays in lung cancer (i.e., two from Dako and two from Ventana medical system) highlighted differences in mean tumor cell and immune cell staining between the assays. Consequently, methods for measuring PDL1 cannot be used interchangeably in clinical practice, thus raising questions on possible technical biases and use for decision-making [28].

This discrepancy is found as well regarding the FDA approval of immune checkpoint inhibitors, because of the great heterogeneity in terms of cut-off [26]. For Nivolumab, during clinical trials different thresholds of PD-L1 expression were tested and ranged from 1% to 10% (i.e., Checkmate studies 017, 025, 057, 066, 067, and 141). All PD-L1 quantifications were performed on tumor cells, and the final choice for a positive cut-off seems to be highly tumor-type dependent.

For Pembrolizumab, during clinical trials different positivity thresholds for PD-L1 expression were tested too, ranging from >1% (Keynote 66) to >50% (Keynote 010 and 024).

All PD-L1 protein expression quantification was performed on tumor cells except for Keynote 006 where PD-L1 was quantified on both tumor cells and in tumor microenvironments. The threshold selection seems to be tumor-type dependent, i.e., high (>50%) for non-small-cell lung cancer (NSCL) and low (>1%) for the other types. For Atezolizumab, during clinical trials different positivity thresholds of PD-L1 expression were tested too, ranging from >1% to >50% [29]. For Durvalumab, a clinical trial NCT01693562 in NSCLC suggests that patient who had detectable levels of PD-L1 expression over 25% on tumor cells may have longer survival [30]. 

These different clinical trials highlight once again the great heterogeneity observed in terms of detectable levels of PD-L1 protein expression and tested material. 

Given the lack of uniformity and positive results in patients defined as negative, the use of levels of PD-L1 protein expression seems difficult in routine practice. Moreover, PD-L1 expression is dynamic and modulated by radiation therapy or chemotherapy [31]. This PD-L1 expression modulation described with radiation therapy and alkylating agents such as platinum-based drugs, is a hope for non-responders patients to immunotherapy as monotherapy [32].

Actually, several drugs can modulate the transcriptional and post-transcriptional regulation of PD-L1. For instance, Lenalidomide, currently used in multiple myeloma patients, down-regulates PD-L1 expression [33]. An in vitro study testing six different drugs (topoisomerase-2 inhibitor, microtubulin inhibitor, CDK (cyclin dependent kinase) 4/6 inhibitor, topoisomerase-1 inhibitor, PI3K-mTOR dual inhibitor, and SRC-3 inhibitor 6) in breast cancer cells demonstrated a PD-L1 mRNA induction in an overwhelming majority of cases [34]. The use of drugs to either up- or down-regulate the expression of PD-L1 is an interesting research path, but this makes interpreting PD-L1 expression in pretreated patients (e.g., with the microtubulin inhibitor or lenalidomide) complicated because it may not reflect the basal expression level.

Technically speaking, tumor quantification of PD-L1 expression requires invasive biopsy procedures in patients. Alternatively, serum soluble PD-L1 quantification is currently being developed. However, no significant association was found between serum or plasma PD-L1 levels and tumoral PD-L1 expression [35]. Importantly, soluble PD-L1 is also present in healthy patients and increases with age [36]. Still, concentrations in soluble PD-L1 are higher in cancer patients, but without a clearly defined positive threshold, soluble PD-L1 will be difficult to interpret, as for PD-L1 expression in tumor cells.

## 5. Riders on the Storm: Tertiary Lymphoid Structures, Tumor-Infiltrating Lymphocyte and Tumor Microenvironment

Tertiary lymphoid structures are developed at an inflammation site, e.g., around a tumor, and from an organization angle they look like lymph nodes. Their role is essential in the adaptive tumor immune response. This inflammatory state at the peripheral of the tumor facilitates the trafficking of lymphocytes, as well as their infiltration, and can also support effective antigen presentation and lymphocyte activation [37]. The presence of tertiary lymphoid structures is associated with a favorable clinical outcome for cancer patients, regardless of the stage of the disease. More particularly, the presence of CD8+ activated T lymphocytes is correlated with a favorable prognosis. Indeed, even if their natural cytotoxic activity is not considered sufficient to be curative [38], lack of lymphocytes is associated with poor response to immunotherapy. The key issue for these lymphocytes is their intratumoral penetration, so their localization has to be close to the tumor and the normalized vasculature [39]. The tumor microenvironment is an interface that promotes tumor growth, which is usually very immunosuppressed. It is characterized by acidic extracellular pH, hypoxia, high interstitial fluid pressure, aerobic glycolysis (a.k.a. the Warburg effect), glutamine addiction, and altered choline-phospholipid metabolism [40]. The neovasculature plays an essential role in the tumorigenic and immunosuppressive capacities of the microenvironment. In fact, tumor vascular stromal cells and endothelial cells decrease the recruitment, adhesion, and activity of T lymphocytes [41]. Beyond carcinogenesis, the anarchic tumor vasculature, as well as the overexpression of pro-angiogenic factors, has been recently implicated in mechanisms of resistance, including those limiting the efficacy of clinically-approved immune checkpoint inhibitors [42]. Tumor vascular normalization seems to be essential in tumor microenvironment regarding T lymphocyte recruitment and activity, decreased inflammatory reaction, and proper drug delivery [43].

Characterization and identification of the tumor microenvironment is essentially performed by immunohistochemical or immunofluorescence detection on biopsy samples and by flow cytometry analysis [44]. It is a pivotal field of research, but the characterization of tumor microenvironments and tertiary lymphoid structures remains complex, especially because their structures are also found in many inflammatory and/or autoimmune diseases.

## 6. Tumor Mutational Burden and Microsatellite Instability: Born to be Wild

The two most sensitive cancer types to immunotherapy are melanoma and NSCLC, which are both characterized by a high mutational tumor burden [45]. Tumor mutational burden and microsatellite instability reflect tumor genomic instability. Production of neo-antigens is the consequence of these mutations. The immune response to these neo-antigens, mediated by CD8+ and CD4+ T lymphocytes, is the basis of immunotherapy. Immune checkpoint inhibitors are highly effective in tumors presenting microsatellite-high instability (MSIH) and DNA mismatch repair-deficient tumors [46]. Consequently in 2018, the FDA approved Pembrolizumab in any advanced solid tumors with those characteristics [47]. 

This agnostic decision goes in the direction of an individualized precision medicine based on the specificities of the tumor for each patient, and does not, anymore, depend on the tumor localization. In the Checkmate 227 study, the first line treatment by two immune checkpoint inhibitors (Nivolumab plus Ipilimumab) showed a significantly longer progression free survival on the tumor mutational burden positive patient (≥10 mutations/Mb) independent of PD-L1 expression in advanced non-small-cell lung cancer [48]. Nevertheless, once again, as with PD-L1 expression the question of defining the right positive threshold is critical with the tumor mutational burden. Some studies have defined a positivity threshold for ≥20 mutations/Mb, i.e., twice as much than the Checkmate 227 study [45].

## 7. Breakout: Cell-free DNA and STING-cGAS Pathway

**Figure 2:**
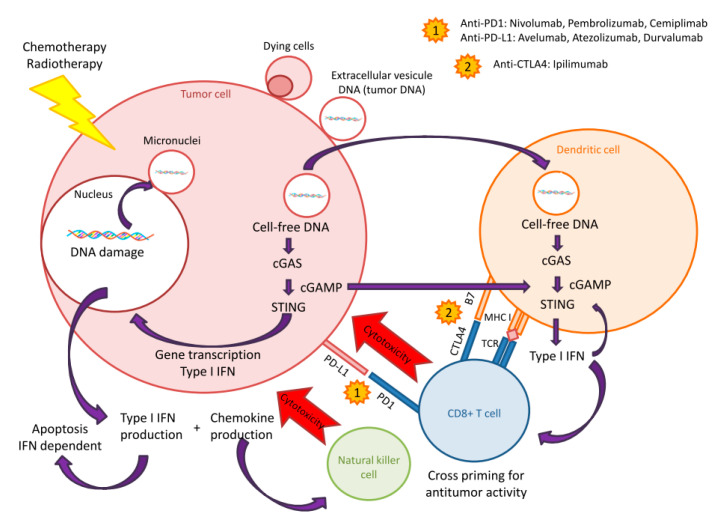
Sting/cGas Pathway.

Cell-free DNA (cfDNA) is an interesting biomarker already used in acute cardiovascular pathologies and as a mortality predictor in myocardial infarction [49] and in inflammatory and autoimmune disease such as severe systemic lupus erythematosus [50]. The role of cfDNA seems to be associated to the STING-cGAS pathway [51]. Discovered in 2009, the STING-cGAS pathway was first described during infection as an effector of type-I interferon (IFN) production [52]. In infectiology, pathogen DNA is first recognized by cytoplasmic cyclic GMP-AMP synthase (cGAS), then the complex binds to stimulator of interferon genes (STING), thus inducing type-I interferon (IFN) and other cytokines’ production [53]. This pathway plays a major role in innate immune response against DNA pathogens. Recently, STING-cGAS role in oncology has been described with immune checkpoint inhibitors [54,55]. In cancer cells, the STING-cGAS pathway is activated by cfDNA produced by two mechanisms. First, the dysregulation of DNA replication in cancer cells by endonucleases leading to cytosolic accumulation in cancer cells. Second, during mitosis damaged DNA produces cytosolic micronuclei which trigger cGAS during their rupture [56]. Accumulation of DNA in cytoplasm is considered as a danger for living cells, thus triggering the innate immune response via the activation of STING-cGAS, regardless of the DNA origin (i.e., exogenous or endogenous), since the production of type-I IFN is a universal response [57]. Type-I IFN plays, indeed, a major role in immune cells’ regulation, especially in Natural Killer (NK) cell proliferation, activation, and antitumor activity. In addition, it enhances the capacity of dentritic cells to cross-present the antigen to the activated CD8+ T lymphocytes [58]. Thus, type-I IFN strengthens innate immunity in patients and, therefore, is likely to increase the efficacy of immune checkpoint inhibitors (Figure 2).

STING-cGAS pathway activation is also found in the senescence phenomenon. In an aging cell, cytosolic cell-free DNA accumulates and the activated STING-cGAS pathway will increase senescence and inflammation [59]. Diagnostic use of cell-free DNA seems to be very promising in clinical oncology. The genome-wide sequencing of cfDNA for non-invasive prenatal diagnostic has been validated in over 100,000 patients, and is currently used now in a routine clinical setting [60].

Indeed, the use of cfDNA as a prognostic marker and monitoring of residual disease is increasing [61]. In addition, cell-free DNA could be used as a surrogate marker for STING-cGAS pathway activation, i.e., to determine immunogenic response to standard therapy such as cytotoxics.

In oncology, different studies showed that cfDNA length in patients are 180 bp, and are thus qualified as short [62] or between 90–150 bp [63]. A total of 180 bp fragments are characteristic for apoptotic cell death, i.e., when cellular chromatin is degraded by a caspase-activated DNase, whereas bigger, 10,000 bp fragments are rather associated with necrotic cell death [64]. The cfDNA from the tumor cell is always shorter than the cfDNA from the non-tumor cells [65]. The size is, therefore, cancer-specific and makes possible to discriminate apoptotic and non-necrotic events in cancer cells. The cfDNA length is, therefore, an important parameter to take into account for STING-cGAS pathway activation. The minimal length for triggering immune reaction is 20–40 bp, and immune optimal response is achieved between 45 and 70 bp [66]. Activation of STING-cGAS pathway and type-I IFN production are, therefore, DNA length-dependent. Otherwise, cfDNA concentration increases in advanced stages of cancer disease, as demonstrated with locally advanced cervical cancer [67] and metastatic colorectal cancer [68]. The cfDNA concentration is, therefore, a dynamic biomarker correlated to disease stage, thus allowing longitudinal monitoring of the tumor and possibly of treatment efficacy [69]. Indeed, both the size and concentrations of cfDNA are linked to IFN expression. The larger the size of the cell-free DNA length, the lower the concentration: 1.67 μg/mL for the DNA fragments of <500 bp, 0.167 μg/mL for the DNA fragments >500 bp and 0.0167 μg/mL for the DNA fragments >2000 bp [66]. For small length cfDNA fragments, concentration is critical because too low concentrations reflect primarily cellular senescence. Of note, small DNA fragments in low concentration do not trigger the STING-cGAS pathway, thus avoiding the auto-inflammation phenomenon [70]. The release from the nucleus of larger DNA fragments leads to the formation of micronuclei in cytoplasm, previously described as playing a key role in the activation of STING-cGAS.

These results suggest that STING-cGAS signaling plays a pivotal role in the intrinsic antitumor immunity and that this pathway should be activated to harness tumor immunity in patients.

As previously described with PD-L1 protein expression, chemotherapy, targeted therapy, and radiation therapy can all activate the STING-cGAS pathway [71].

For instance, radiation therapy can increase damaged DNA and cytosolic accumulation via STING-cGAS activation [72]. This could partly explain the use of radiation therapy for its ability to enhance responses to immunotherapy, since ionizing radiation-mediated tumor regression depends on type-I IFN and the adaptive immune response [73].

Regarding cytotoxics, the alkylating agent Cisplatin has been described as a powerful immunomodulating drug. Cisplatin boosts the immunogenicity of the tumor, i.e., by increasing the expression of Major Histocompatibility Complex class I (MHC I) at tumor cell surface, thus facilitating recognition by activated CD8+ T lymphocyte. Cisplatin also upregulates the STING-cGAS pathway [74]. These immunomodulatory properties could explain the synergistic effects shown between cisplatin and immune checkpoint inhibitors. Antitumor efficacy with this combination could be explained by the increased number in tumor-infiltrating CD8+ and CD4+ T lymphocytes [75]. Topoisomerase II inhibitors (e.g., doxorubicin, etoposide and epirubicin) were also described as modulating the STING-cGAS pathway and cfDNA [76]. In a study of cfDNA kinetics, the peak of DNA seems to be the highest 24 h after exposure to doxorubicin and epirubicin in breast cancer models [77]. These dynamics was also observed, in the same study, with 5-Fluorouracil. This time window could correspond to a maximum STING activation and calls for administrating immune checkpoint inhibitors 24 h after that of cytotoxics, thus suggesting a possible sequence-effect when combining drugs with immunotherapy. 

Some targeted therapy, such as Poly ADP Ribose Polymerase (PARP) inhibitors seems to modulate the STING-cGAS pathway as well [78]. PARP is implicated in the DNA repair process, for which inhibition leads to an accumulation of cytosolic DNA, further activating the STING-cGAS pathway [79]. PARP inhibitors induce STING-dependent antitumor immunity [80]. This induced immunity is independent of the BRCA status of the patients [81] as demonstrated in gynecological cancers [80]. The role of PARP inhibition in cytosolic DNA accumulation was also described in prostate cancer [82]. Immunomodulating features with PARP inhibitors have been also demonstrated in NSCLC [83]. In vivo, small-cell lung cancer models showed that PARP response to damaged DNA increases PD-L1 protein expression, and increased cytotoxic T-cell infiltration [84]. These two mechanisms helped to boost antitumor effect of immune checkpoint inhibitors eventually.

Regarding immunotherapy, the STING-cGAS pathway has been identified as essential for the antitumoral effect of immune checkpoint inhibitors. For instance, STING-deficient mice are refractory to the antitumor effects of PD-L1 [85]. Conversely, direct administration of the STING or cGAS agonist shows an antiproliferative effect, and the STING agonist further reverses resistance to anti-PD1 [86]. Elsewhere, cGAS administrated as nanoparticles increases production of type-I IFN, however, with no impact on tumor growth in the absence of immunotherapy [87]. This last study underlines the major role of STING-cGAS pathway on type-I IFN production and T lymphocyte activation but also the essential role of immune checkpoint inhibitors to translate this into therapeutic effect. Link between STING-cGAS pathway activation and immune checkpoint inhibitor efficacy has been demonstrated in several studies [88]. In ovarian cancer mouse models, overexpression of STING-cGAS upregulates PD-L1 protein expression [74]. This pathway appears to be an interesting target for potentiating the immunotherapy effect. Numerous clinical trials are currently underway studying agonist STING combining immune checkpoint inhibitors or in monotherapy from pre-clinical to phase 3 study [89].

Finally, as previously mentioned, the STING-cGAS pathway plays a major role in activated T lymphocytes by increasing the production of several chemokines. Among them, CCL5 and CXCL10 are known as being chemotactic for T lymphocytes, allowing tumoral accumulation and increasing tertiary lymphoid structures’ formation [90]. STING further activates dendritic cells and initiates the priming of T lymphocytes [91]. Study of the STING-cGAS pathway activation by cfDNA is, therefore, an indirect way of studying the tumoral microenvironment and the tumor-infiltrating lymphocyte [92].

## 8. cfDNA and STING-cGAS Pathway Activation: Stairway to Heaven or Highway to Hell?

The cfDNA-mediated activation of the STING-cGAS pathway seems, therefore, to be a promising way to optimize immune checkpoint inhibitor therapy; however, this pathway is a double-edged sword [93]. Beyond the antitumoral effect of STING-cGAS, it also plays a major role in carcinogenesis, particularly via inflammation activation, autoimmune response, and direct tissue toxicity [71]. In carcinogenesis, MYD 88 (molecule myeloid differentiation factor 88) signaling seems to play a major role in cancer development via cytokine, chemokine, and growth factor production. In functional STING mice exposed to DMBA (a polyaromatic hydrocarbon known as carcinogenic agent), cutaneous skin tumors with pro-inflammatory cytokine production and phagocytic infiltration were observed [94]. Conversely, deficient STING mice did not develop such skin tumors post DMBA exposure, suggesting the role STING could play in skin cancer development [95]. Conversely, in some cancer types such as melanoma or colon cancer, the STING-cGAS pathway can be impaired by loss-of-function mutation or epigenetic silencing of the STING-cGAS promoter regions [96]. In this case, quantification of cell-free DNA could not be associated to an upregulation of STING-cGAS. Furthermore, in tongue squamous cells induced by human papillomavirus models, STING-cGAS activation eases T Regs infiltration and could, therefore, have a negative impact [97].

The cfDNA, itself, can be a confounding factor. As previously underlined, cfDNA is more important in cancer patients, and is not always synonymous of inflammatory situations. The cfDNA reflects genome plasticity, leading to a physiological and autonomous process of cells for its elimination [59]. Half-life of cfDNA should also be considered; it varies between 15 min and few hours [98], thus raising questions about appropriate sampling time for decision-making.

## 9. Discussion: Shine a Light

Immunotherapy and more specifically, immune checkpoint inhibitors, have fueled huge hope in oncology. However, the clinical results in terms of survival have failed to meet the initial expectations because only a minority of patients show long term survival, in a minority of cancer disease such as melanoma, NSCLC, head, neck, and kidney cancers. Consequently, combinatorial regimens now turned to as the rule, and not anymore, the exception with immunotherapy, in an attempt to harness tumor immunity prior to administrating immune checkpoint inhibitors. This strategy is expected to transform promising and breakthrough pharmaceutical innovations into meaningful survival in patients. The main difficulty when using immune checkpoint inhibitors is the complexity of their mechanisms of action which cannot be reduced to PD-L1, PD1, or CTLA4 inhibition anymore as once thought, but require as well, an adequate tumor micro-environment enriched with activated lymphocytes with little T Regs or MDSC activity. In this respect, using canonical cytotoxics is an appealing strategy to increase immunogenic cell death while down-regulating immunosuppressive cells [99]. However, achieving such immunomodulating features requires fine tuning since they are much probably drug- and dose-dependent [100]. For instance, in several non-clinical studies combining anti-PD1 or anti-PDL1 with anti CDK 4/6 or anti-OX40 drugs, it has been demonstrated that even slight changes in scheduling are likely to dramatically change the response to the combinations [101]. The same phenomenon has been observed as well when combining immune checkpoint inhibitors with radiation therapy [102]. All these studies call for a comprehensive understanding of the exact dynamics of tumor reengineering when drugs are used to harness immunity and expected to yield synergistic effect with immunotherapy. Indeed, capturing this exact dynamic is a tricky but critical issue, because it could provide valuable information on the best dosing, timing, and sequencing, especially when combining cytotoxics with immune checkpoint inhibitors. Unfortunately, as of today current combinatorial strategies remain empirical and concomitant dosing is frequent. Consequently, many clinical trials have failed to yield meaningful results in terms of prolonged survival [103]. Notably, as previously explained, baseline PDL1 expression levels cannot help determining the optimal modality of combination between immune checkpoint inhibitors and associated treatments such as chemotherapy. The same observation can be made with tumor burden, medical records, microbiota features, or any of the numerous parameters tested so far as putative predictive markers with immunotherapy. This calls for using more dynamic markers better reflecting real-time changes in the tumor micro-environment such as immunogenic cell death. Because as seen before, STING-cGAS is a critical signaling pathway associated with response to immunotherapy and that release of cfDNA, especially the shorter ones, activates this pathway, blood quantification of cfDNA could be a convenient surrogate to monitor STING-cGAS activation. This could help predicting the best timing to use immune checkpoint inhibitors next. As previously stated, cfDNA seems to be the only biomarker whose kinetics would allow to define this optimal window, especially with a combinatorial regimen. Indeed, peak of cell-free DNA is dependent upon the tumor itself but also by the concomitant use of other anticancer therapies such as chemotherapy, radiation therapy, but also oral targeted therapies. By discriminating large (i.e., >10,000 bp) cfDNA associated with necrosis from the short (i.e., 180 bp) ones associated with tumor apoptosis with immunogenic response via STING-cGAS, it paves the way for a qualitative and quantitative monitoring of cancer patients. 

In addition, quantification of cfDNA is achieved by liquid biopsy, thus facilitating its implementation and allowing repeated and longitudinal measures throughout time. As a comparison, immunomonitoring provides valuable information on activated T lymphocytes or T Regs (i.e., to assess the impact of chemotherapy on the immune system,) but this can only be done after tumor biopsy, thus limiting its repeated use in routine patients. 

**Figure 3:**
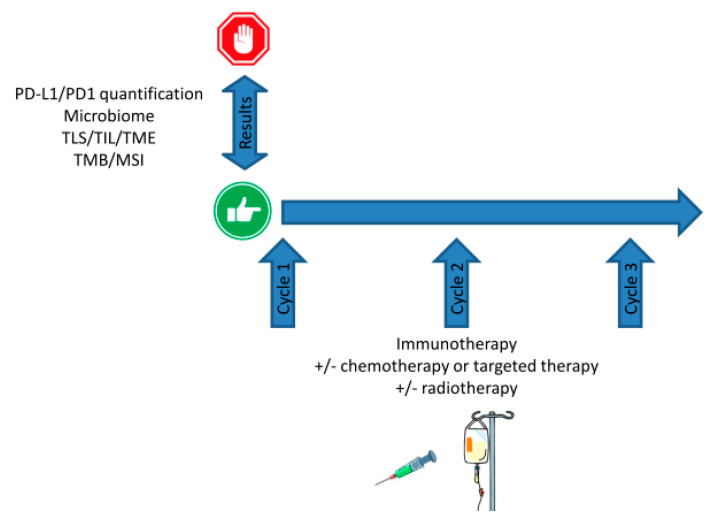
Current use of biomarker prior to setting up combinatorial immunotherapy. Upfront testing helps to determine the Go/No Go by predicting the odds of success. However, no longitudinal monitoring is currently feasible and basal levels have to be considered as granted.

To date, current biomarkers such as TMB, MSIH, or PD-L1 expression levels are mostly used prior to therapy in a binary, Go/No-Go, fashion. Indeed, despite the current vagueness for defining positivity threshold, PDL1 expression is a green light for using immune checkpoint inhibitors, as MSIH stats for pembrolizumab (Figure 3). Once treatment has started, monitoring these markers does not allow tuning, nor dosing, scheduling, or sequencing should combinatorial regimen be administrated, and evaluation for response is performed several cycles later.

**Figure 4:**
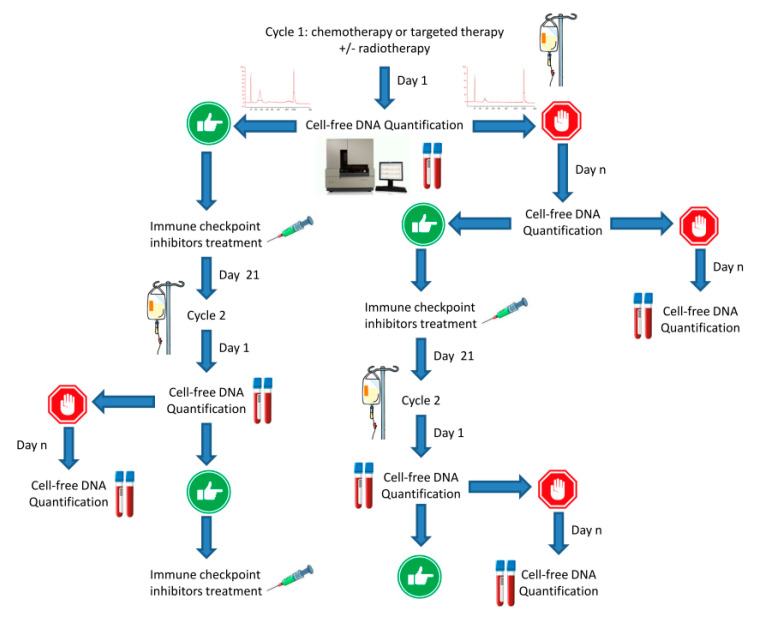
Proposed strategy for refining combinatorial immunotherapy. After that standard treatment is given, longitudinal monitoring of cell-free DNA helps to determine the best timing for further administering immune checkpoint inhibitors. Rather than pre-defined dosing, longitudinal monitoring allows customized treatment throughout time.

Conversely, monitoring cfDNA is a dynamic strategy. The cfDNA can also be a Go to start immunotherapy. In case of absence of a DNA peak, this calls for starting for treatments such as neo-adjuvant chemotherapy or radiation therapy expected to trigger immunogenic cell death—monitoring cfDNA could thus help to determine the best time-window to start immunotherapy with respect to changes in tumor immunity (Figure 4). By doing so, the current concomitance of treatments should not be longer the rule as immune checkpoint inhibitors administration would be more wisely guided by a real-time biomarker. With respect to the ever-increasing drug-costs in oncology, developing novel strategies to optimize treatment efficacy and treatment cost-effectiveness is now critical. Rather than relying on a basal biomarker, longitudinal monitoring would allow fine tuning of the therapy, i.e., by stopping a therapy which is doomed to fail, before imaging reveals treatment failure. Unlike costly pan-genomic analysis of tumors or microbiota, cfDNA could be a cheap, rapid, non-invasive, and convenient way to check whether immunotherapy is likely to yield clinical benefit or not.
